# Kinematic movement and balance parameter analysis in neurological gait disorders

**DOI:** 10.1186/s13036-023-00398-w

**Published:** 2024-01-15

**Authors:** Chuh-Hyoun Na, Hannah Lena Siebers, Julia Reim, Jörg Eschweiler, Frank Hildebrand, Hans Clusmann, Marcel Betsch

**Affiliations:** 1 Department of Neurosurgery, University Hospital RWTH Aachen, Pauwelsstr. 30, Aachen, 52074 Germany; 2 Department of Orthopaedic, Trauma and Reconstructive Surgery, University Hospital RWTH Aachen, Aachen, Germany; 3 Department of Trauma and Orthopedic Surgery, University Hospital Erlangen, Friedrich-Alexander-University Erlangen-Nürnberg (FAU), Erlangen, Germany

**Keywords:** IMU, Gait analysis, Pedobarography

## Abstract

**Background:**

Neurological gait disorders are mainly classified based on clinical observation, and therefore difficult to objectify or quantify. Movement analysis systems provide objective parameters, which may increase diagnostic accuracy and may aid in monitoring the disease course. Despite the increasing wealth of kinematic movement and balance parameter data, the discriminative value for the differentiation of neurological gait disorders is still unclear. We hypothesized that kinematic motion and balance parameter metrics would be differently altered across neurological gait disorders when compared to healthy controls.

**Methods:**

Thirty one patients (9 normal pressure hydrocephalus < NPH > , 16 cervical myelopathy < CM > , 6 lumbar stenosis < LST >) and 14 healthy participants were investigated preoperatively in an outpatient setting using an inertial measurement system (MyoMotion) during 3 different walking tasks (normal walking, dual-task walking with simultaneous backward counting, fast walking). In addition, the natural postural sway of participants was measured by pedobarography, with the eyes opened and closed. The range of motion (ROM) in different joint angles, stride time, as well as sway were compared between different groups (between-subject factor), and different task conditions (within-subject factor) by a mixed model ANOVA.

**Results:**

Kinematic metrics and balance parameters were differently altered across different gait disorders compared to healthy controls. Overall, NPH patients significantly differed from controls in all movement parameters except for stride time, while they differed in balance parameters only with regard to AP movement. LST patients had significantly reduced ROMs of the shoulders, hips, and ankles, with significantly altered balance parameters regarding AP movement and passed center-of-pressure (COP) distance. CM patients differed from controls only in the ROM of the hip and ankle, but were affected in nearly all balance parameters, except for force distribution.

**Conclusion:**

The application of inertial measurement systems and pedobarography is feasible in an outpatient setting in patients with different neurological gait disorders. Rather than defining singular discriminative values, kinematic gait and balance metrics may provide characteristic profiles of movement parameter alterations in the sense of specific ´gait signatures´ for different pathologies, which could improve diagnostic accuracy by defining objective and quantifiable measures for the discrimination of different neurological gait disorders.

**Trial registration:**

The study was retrospectively registered on the 27th of March 2023 in the ‘Deutsches Register für Klinische Studien’ under the number DRKS00031555.

## Introduction

Gait deviations leading to changes in spatiotemporal, kinematic, and kinetic gait parameters are common in neurological patients and have a significant influence on everyday life [1, 2]. Due to the high impact of walking abilities on participation in daily activities and quality of life, gait recovery in neurological patients is essential [3], and socioeconomically highly relevant in public health. However, a premise for the effective treatment of gait disorders is to correctly identify the cause, which can be obscured by overlapping symptoms of different diseases or coinciding pathologies. As gait disorders are at present mainly classified based on clinical observation, they are difficult to objectify or quantify, and clinical assessment might be even more challenging for non-experts of neurological movement disorders.

Given the poor reliability and subjectivity of observer-dependent gait assessments, instrumented gait analysis has been implemented to objectively measure and compare movement parameters [3, 4]. Currently, a whole range of different measurement techniques and systems for non-invasive motion analysis are available. Concerning neurological gait disorders, mainly spatiotemporal movement parameters have been investigated so far, while kinematic movement analysis is still rare in this patient group. Based on spatiotemporal gait parameters detected with a pressure-sensitive carpet, Pradhan et al. were successful in the automated classification of several neurological disorders by different gait patterns [5]. Nevertheless, such pressure-sensitive carpets, built-in force plates, instrumented treadmills, or complete optical-electronic motion tracking systems are more or less limited to laboratory use only, which is often associated with long data acquisition and analysis times. In contrast, inertial measurement units (IMUs) allow a portable application and mobile data acquisition, enabling measurements in a real-world environment. This is of special relevance, as Kuruvithadam et al. previously showed, that movement patterns of patients significantly differ between real-world and laboratory environments [6].

Kinematic motion and balance measurement systems may support the diagnostic procedure and treatment decision-making while monitoring the outcome during rehabilitation [3, 7]. Gait analysis technologies may help to further improve diagnostic accuracy, especially in geriatric patients, which are difficult to assess due to comorbidities and cognitive impairment. With regard to NPH, at present, only an estimated 10–20% of all patients are correctly diagnosed [6, 8], although it constitutes one of the few effectively treatable causes of dementia and progressive immobility. While NPH is characterized by the cardinal symptoms of gait disturbance, dementia, and urinary incontinence, these symptoms have only a low specificity, and may as well occur in other neurodegenerative disorders [8, 9]. If however NPH is correctly diagnosed in the early stage of the disease, its symptoms can still be reversed [8, 9].

Besides NPH, lumbar stenosis (LST) and cervical spondlytic myelopathy (CM) are commonly encountered in the neurosurgical setting as these conditions potentially responsive to surgical intervention, and improving diagnostic accuracy might dramatically influence treatment decisions and the individual functional outcome. While data on instrumented gait analysis applied for the non-invasive evaluation of neurological gait disorders is steadily increasing, the discriminative value for distinguishing different pathologies remains however unclear.

Regarding NPH, wearable motion sensors for gait analysis have previously been used to characterize motion and postural response parameters in NPH patients compared to healthy controls [7–10], or to evaluate effects of therapeutical intervention pre- and post spinal tap, or pre- and post shunt surgery [11–13]. The relevance of environmental factors for the evaluation of gait parameters has been shown not only in healthy controls [14], but as well in this patient group [9, 12, 15]. Likewise, patients with CM have repeatedly been characterized using kinematic gait or balance parameter analysis by comparison to healthy controls [16–21], or pre- and postoperatively for the evaluation of treatment effects [18, 20–23]. In LST patients, kinematic gait analyses showed differences in spinal and pelvic movement, and aided to quantify movement deviations compared to healthy controls [24, 25]. Instrumented gait analysis was furthermore used to correlate movement alterations to back pain [26], and to objectify treatment effects postoperatively [27–29] in LST patients.

There is however a lack of data using kinematic movement and balance parameter analyses to actually distinguish different gait disorders. Comparison of different gait disorders across different studies is highly limited due to the lack of standardization regarding measurement protocols, choice of parameters, and type of data analyses. By using the same standardized measurement setup and measurement protocol for different patient groups within the same study, we therefore investigated, whether inertial measurement systems combined with pedobarography provide objective measures for the discrimination of different gait pathologies, using healthy controls as a common reference frame. We evaluated the applicability of an inertial measurement system in an outpatient setting, and analyzed balance and gait parameters in patients with NPH, LST, and CM. We hypothesized that patterns of kinematic motion and balance parameters would be differently altered across different patient groups when compared to healthy controls.

## Material and methods

### Participants

To analyze different neurological gait disorders, thirty-one patients with NPH, CM, and LST were investigated preoperatively in the neurosurgery department of a university hospital center. 14 healthy subjects were included as controls. Only subjects without musculoskeletal or (in the case of patients: additional) neurological or psychiatric disorders potentially impeding walking or standing abilities were enrolled in the study. Exclusion criteria were age < 18 years and pregnancy. Only subjects who gave their informed written consent were included. The study was approved by the local ethics committee (EK 148–18) and conducted according to GCP principles and the Declaration of Helsinki. Motion parameters were analysed during three different walking tasks (15 m walking with self-selected comfortably and speed, dual-task walking with backward counting, and fast walking) and two conditions during static standing (with the eyes opened and closed). The different walking and balance tasks were chosen to analyse different effects on movement and balance parameters [30, 31]. The anthropometric data of the tested groups are presented in Table 1.

**Table 1:** Anthropometric data of the study participants

Group	N (female)	Age [years]	Height [cm]	Weight [kg]	BMI [kg/m^2^]
NPH	9 (4)	76 ± 5.3	168 ± 7.4	80 ± 11.6	28.4 ± 3.7
CM	16 (4)	58 ± 9.9	174 ± 9.2	81 ± 19.2	26.4 ± 4.7
LST	6 (1)	69 ± 10.2	180 ± 3.1	97 ± 11.8	29.8 ± 3.7
Control	14 (6)	34.2 ± 11.8	174 ± 8.2	73 ± 18.7	24.0 ± 4.0

### Measurement setup

**Fig. 1:**
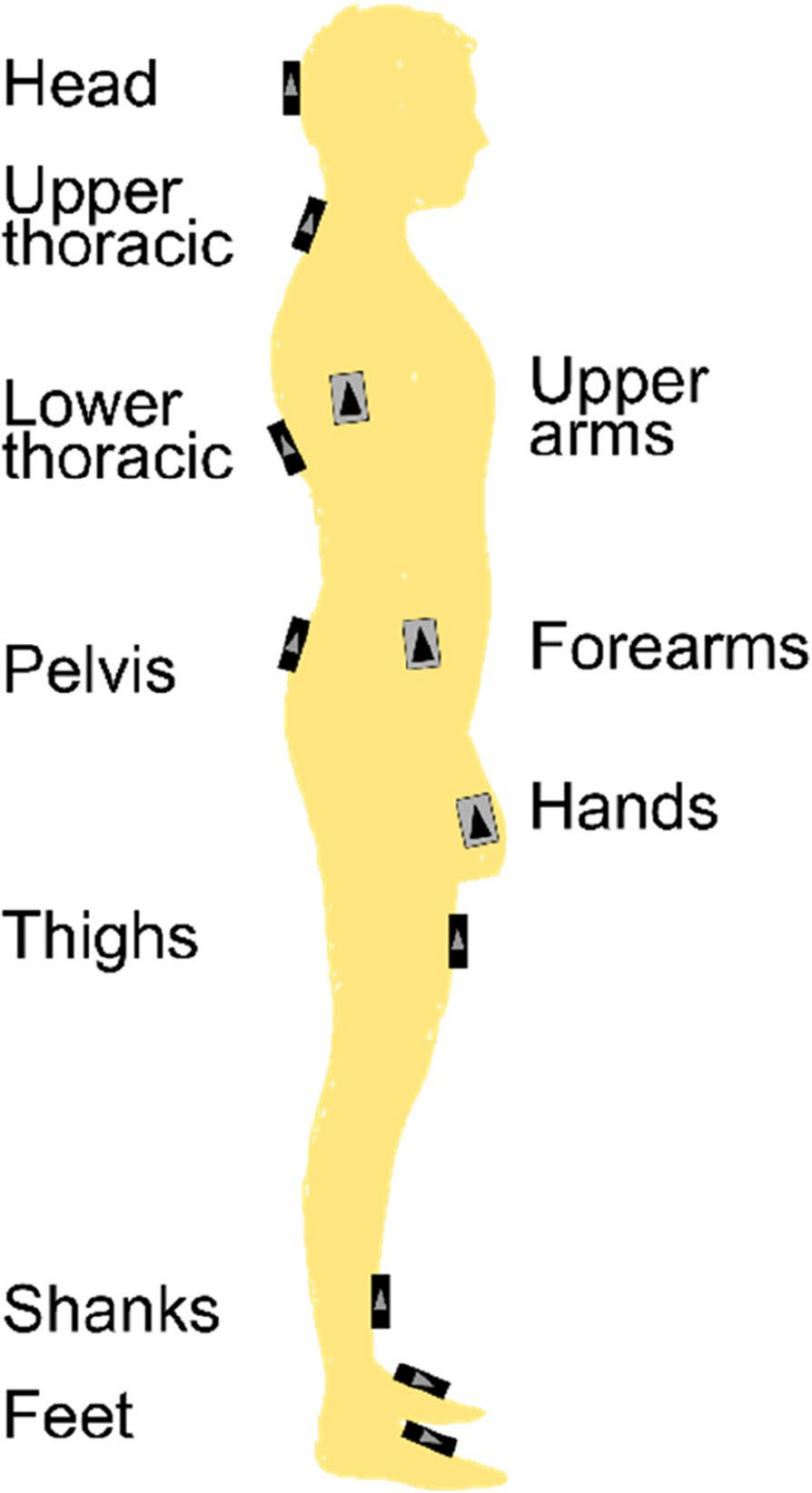
Sensor placement for the MyoMotion IMUs

Kinematic motion data during ground-level walking was measured with an IMU system MyoMotion (Noraxon U.S.A. Inc., Scottsdale, USA). Sixteen IMUs were positioned on the participants according to the user manual (Fig. 1).

After sensor placement, the measurements began with the calibration of the IMU system. For the calibration, the participants stood upright with extended knees and arms hanging on the sides of the body (defined as joint angles = 0°). After calibration, the participants walked at a comfortable self-selected velocity on a straight walkway for 15 m. They were measured with the IMUs on the marked pathway for 10 m in the middle part (without the first and last two steps) to avoid any start or stop movements which ensures that confounding influences on gait parameters were minimised. The IMUs used are ideal for this kind of assessments since they have low drift and offset, making them suitable for static measurements over longer periods of time [13, 32]. After a short break, on the way back, the participants were instructed to count backward from 50 while walking as normally as possible [31]. In the third trial, after a short break, the participants were instructed to increase the walking velocity self-determined if possible [30]. After the motion analysis with the MyoMotion system, a pedobarographic measurement was performed with the DIERS Formetric system (DICAM version 3, DIERS International GmbH, Schlangenbad, Germany). The balance of the participants was analysed during standing, first with the eyes opened, then closed. During this test, the patients stood upright with parallel-positioned feet. The arms were positioned relaxed beside the body and the head was oriented upright with eyes straight ahead. The balance parameters were automatically calculated by the system during the 10 s measurement duration. No repetitions were performed.

In total, each participant was analysed during 3 walking trials and two standing trials.

### Data processing

During walking, anatomical joint angles, segment orientation angles, and accelerations were measured in 3 dimensions at a sampling frequency of 100 Hz. For stride detection, the MyoResearch contact mode was used, and strides were interpolated to 100 points and normalized to one gait cycle. The mean of 5 strides for each trial was calculated. Stride detection is a commonly used method for data reduction, interpretation, and comparison [33]. The data was exported to MATLAB for further calculations (MathWorks R2019a, MathWorks, Natick, MA, USA). The average of right and left side data was calculated. For statistical evaluation, maxima, minima, and range of motion (ROM in [ °]) during one gait cycle, and the stance and swing phases were calculated.

We usded spider charts to present the multidimensional results. While spider charts are two-dimensional, severeal series of values can be plotted over multiple quantitative variables. These dimensions are usually quantitative and typically range from zero to a maximum value. Each dimension’s range is normalized to one another so that the length of a line from zero to a dimension’s maximum value will be the same for every dimension.

### Statistical analysis

To detect significant gait deviations, a mixed model ANOVA was calculated. The mixed model ANOVA is one of the most important forms of variance analysis and is mainly used in clinical and medical settings. The model was prepared for the parameters of the gait analysis: shoulder ROM in the sagittal plane, hip ROM in the sagittal plane, hip ROM in the transversal plane, ankle maximum plantarflexion, and ankle ROM in the transversal plane during one gait cycle; in addition, the knee ROM in the sagittal plane during the stance phase, and the stride time. As well as for the parameters of the pedobarographic data: force distribution anterior/ posterior (AP), AP movement, lateral movement, and passed COP distance. The diagnoses (NPH, CM, LST, or healthy) were analyzed as between-subject factors, and the trials (walking, dual-task, fast walking or open, closed eyes) as within-subject factors, in addition to their interactions. Our intermediate subject factor (between) would be the group affiliation (control group/ experimental group). The within-subject factor (within) would be time, as measured as the dependent variable several times in the same person. Possible differences between the two groups were analysed by Bonferroni Post-Hoc tests. Sphericity was analyzed with the Mauchly-Test, and, in case of violations, the Greenhouse–Geisser (GG, < 0.75) or Huynh–Feldt (HF, > 0.75) correction was used. The Levene-Test was used to test the homogeneity of variance, in case of violations, log transformation in terms of the natural logarithm (ln) has been applied. For interpretation, the calculated differences and confidence intervals were back-transformed to their original scale. The level of significance was defined as 0.05.

All statistical calculations were prepared with the help of IBM® SPSS Statistics (IBM® SPSS Statistics v. 25, IBM Cooperation, Chicago, Illinois, USA).

## Results

Statistical analysis showed significant differences across groups (NPH, CM, LST, controls) for all presented gait and balance parameters, except for *stride time* as well as for *force distribution (AP)* (Table 2). Parameters significantly differed depending on the trial (walking, dual-task walking, fast walking, and pedobarography with the eyes opened and closed), except for *ankle maximum plantarflexion during one gait cycle* (Table 2). There was a significant interaction effect between diagnosis and trial groups for the parameter *force distribution (AP)* (Table 2).

**Table 2:** Statistical results of the mixed ANOVA

Parameter	Diagnosis effect		Trial effect		Interaction effect	
	*p*	η^2^ (%)	*p*	η^2^ (%)	*P*	η^2^ (%)
**Shoulder ROM sagittal plane during one gait cycle**	**.001**	38.3	** < .001(GG)**	37.1	.055(GG)	17.1
**Hip ROM sagittal plane during one gait cycle**	**.001**	37.9	** < .001(GG)**	55.6	.058(GG)	16.6
**Hip ROM transversal plane during one gait cycle**	**.001**	38.6	** < .001**	19.9	.050	16.0
**Knee ROM sagittal plane during the stance phase**	**.009**	27.0	**.002(HF)**	16.2	.165(HF)	11.8
**Ankle maximum plantarflexion during one gait cycle**	** < .001**	44.9	.058(HF)	8.2	.658(HF)	5.1
**Ankle ROM transversal plane during one gait cycle**	**.003**	31.9	**.009**	12.6	.406	8.2
**Stride time**	.058	18.5	** < .001(GG)**	69.0	.126(GG)	13.2
**Force distribution (AP)**	.255	9.3	**.004**	18.6	**.016**	22.1
**AP movement (ln)**	**.001**	34.7	**.001**	25.6	.219	10.1
**Lateral movement (ln)**	**.033**	19.0	**.045**	9.5	.324	8.0
**Passed COP distance (ln)**	**.004**	27.1	**.001**	24.2	.284	8.8

*AP* Anterior/posterior, *GG* Greenhouse–Geisser, *HF* Huynh–Feldt

Log transformations in terms of the natural logarithm (ln) have been applied in case of violation against the homogeneity of variance. Significant values are given in bold

## Kinematic motion parameter analyses during walking

Further pairwise comparisons of different patient groups with healthy controls showed a significant decrease in the *shoulder ROM in the sagittal plane during one gait cycle,* as well as in the *hip ROM in the sagittal plane during one gait cycle* in LST and NPH patients (Table 3). *Hip ROM in the transversal plane during one gait cycle* was significantly decreased in NPH and CM patients compared to the control group. *Knee ROM in the sagittal plane during the stance* phase was significantly reduced only in NPH patients, while *ankle maximum plantarflexion* and *ankle ROM in the transversal plane during one gait cycle* was significantly reduced in all patients compared to controls (Table 3).

**Table 3:** Pairwise comparison of walking tasks

		**Shoulder ROM sagittal plane during one gait cycle**	**Hip ROM sagittal plane during one gait cycle**	**Hip ROM transversal plane during one gait cycle**	**Knee ROM sagittal plane during the stance phase**	**Ankle maximum plantarflexion during one gait cycle**	**Ankle ROM transversal plane during one gait cycle**	**Stride time**
**NPH vs Control**	P	**.002**	**.004**	** < .001**	**.020**	**.001**	**.005**	.155
	Mean Difference [CI]	**-13°** **[-23;-4]**	**-10°** **[-18;-3]**	**-8°** **[-14;-3]**	**-9°** **[-17;-1]**	**-12°** **[-20;-4]**	**-9°** **[-15;-2]**	108 ms [-22; 238]
**CM vs Control**	P	.083	1.000	**.017**	1.000	** < .001**	**.043**	.195
	Mean Difference [CI]	-8° [-16;1]	-.9° [-7;6]	**-5°** **[-10;1]**	.1° [-7;7]	**-11°** **[-18;-4]**	**-6°** **[-12;0]**	89 ms [-23; 201]
**LST vs Control**	P	**.007**	**.036**	.160	1.000	**.004**	**.044**	.231
	Mean Difference [CI]	**-13°** **[-23;-3]**	**-9°** **[-17;0]**	-5° [-10;1]	-4° [-13;5]	**-12°** **[-20;-3]**	**-7°** **[-15;0]**	109 ms [-33; 251]
**Walking vs Dual Task**	P	.166	1.000	1.000	**.026**	1.000	1.000	** < .001**
	Mean Difference [CI]	-2° [-4;0]	.1° [-1;1]	.3° [-1;2]	**2°** **[0;4]**	-.1° [-1;1]	-.5° [-2;1]	**-123 ms** **[-182; -64]**
**Walking vs Fast Walking**	P	** < .001**	** < .001**	**.005**	1.000	.122	**.015**	** < .001**
	Mean Difference [CI]	**-7°** **[-10;-3]**	**-5°** **[-7;-3]**	**-2°** **[-3;0]**	-.1° [-1;1]	-1° [-3;0]	**-2°** **[-4;0]**	**144 ms** **[107; 181]**
**Fast Walking vs Dual Task**	P	** < .001**	** < .001**	**.003**	**.013**	.269	.086	** < .001**
	Mean Difference [CI]	**5°** **[2;8]**	**5°** **[3;7]**	**2°** **[1;3]**	**2°** **[0;4]**	1° [-1;3]	2**°** [0;3]	**-267 ms** **[ 327; 207]**

Significant values are given in bold

Pairwise comparisons between patient groups showed a significantly reduced *hip ROM in the sagittal plane during one gait cycle* (mean difference of 9 degrees, Cl [-17;-2], *p* < 0.008), and in the *knee ROM in the sagittal plane during the stance phase* (mean difference of 9 degrees, Cl [-17;-1], *p* < 0.014) for NPH patients compared to CM patients.

**Fig. 2:**
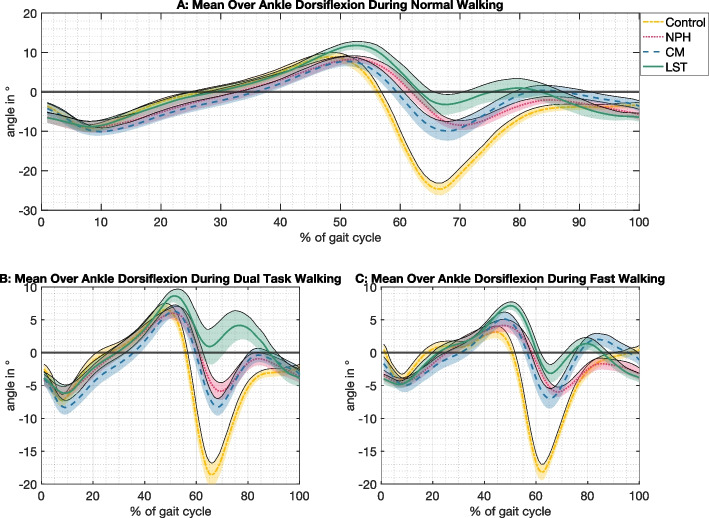
Mean ankle dorsiflexion over one gait cycle for all participant groups

**Fig. 3:**
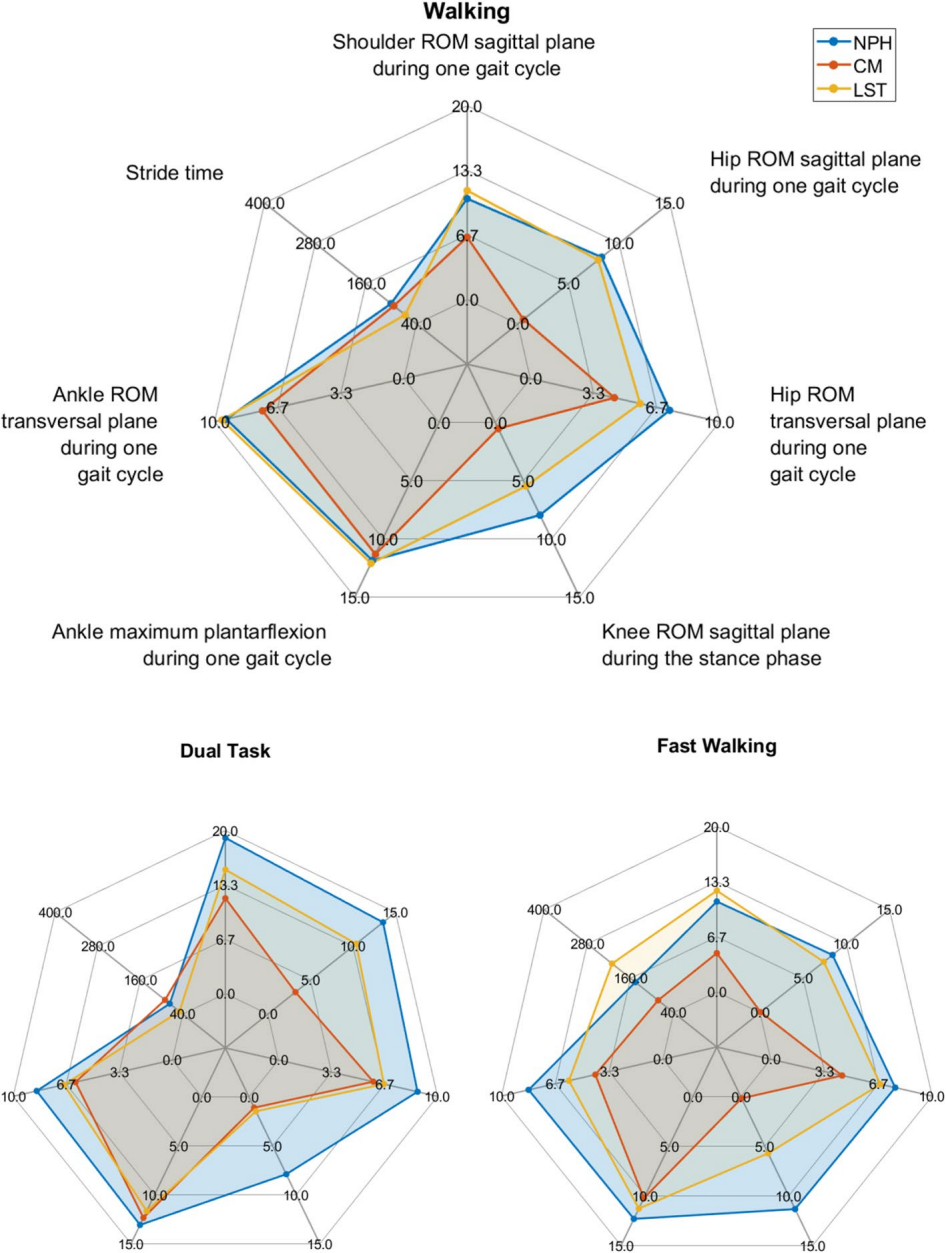
Spider plot of the mean deviations of different patient groups relative to healthy controls. Legend: On each axis, the deviation for one parameter is shown, with kinematic angles in degree and stride time in milliseconds. The center represents no deviation from controls. The mean deviation is presented for each patient group for each walking task separately

Comparison of different walking conditions showed significant changes for *knee ROM in the sagittal plane* and *stride time* when comparing walking versus dual-task. Comparing walking versus fast walking led to significant alterations of all parameters except for *knee ROM in the sagittal plane* and *ankle maximum plantarflexion*. Comparison of fast walking versus dual-task walking had effects on all parameters except for *ankle maximum plantarflexion* (Table 3). As an example, Fig. 2 shows the ankle plantar- and dorsiflexion in the sagittal plane over one gait cycle across different groups and conditions. Figure 3 shows the different effects of walking trials for each patient group relative to healthy controls.

**Fig. 4:**
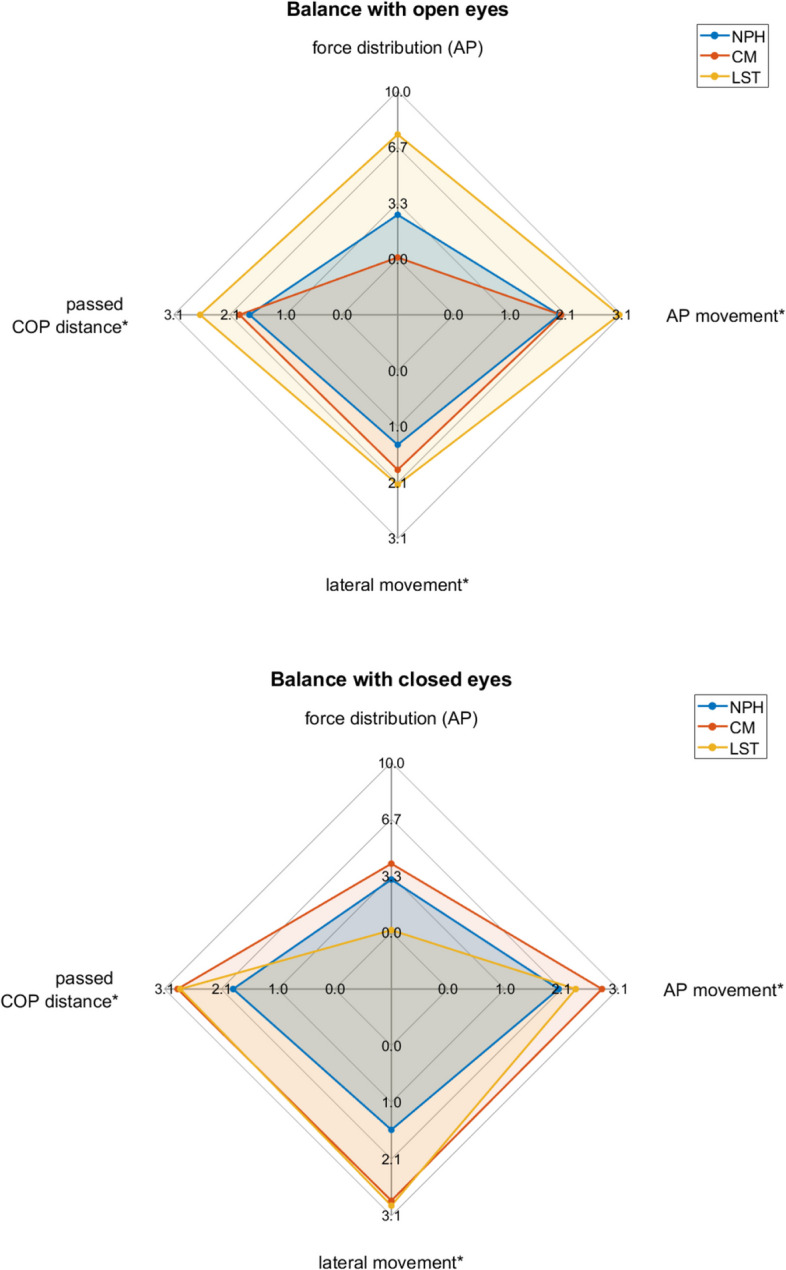
Spider plot of the mean deviations of different patient groups relative to healthy controls. Legend: On each axis, the deviation for one parameter is shown, with movement and distance in millimeters and force distribution in percent. The center represents no deviation from controls. The mean deviation is presented for each patient group for each balance task separately

While NPH and LST patients showed similar profiles during walking (Fig. 4), relative between-group differences seemed to become apparent during the fast walking and the dual task condition. CM patients seemed to be the least impaired in all motion parameters during walking. Unlike the other two patient groups CM patients even showed a relative improvement in movement parameters during the fast walking condition.

### Pedobarography

**Fig. 5:**
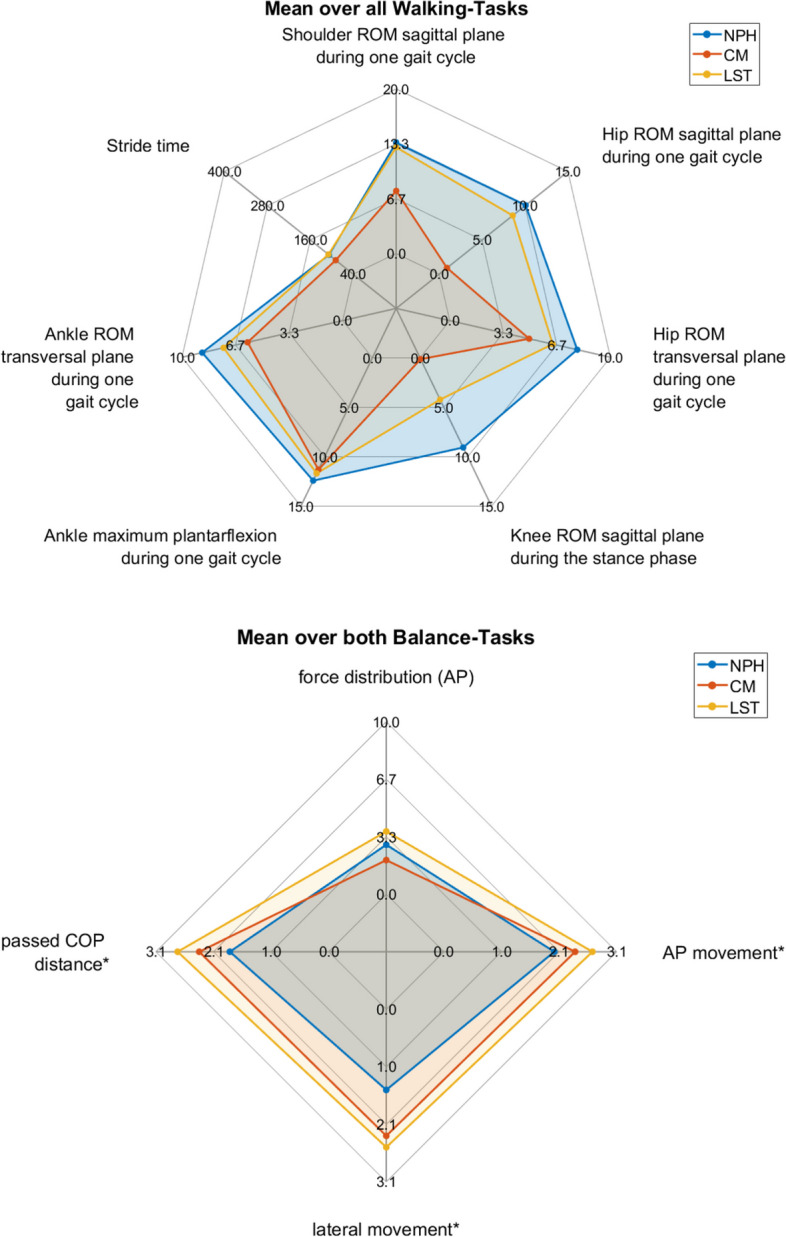
Spider plots of mean deviation of patients across walking (top) and balance tasks (bottom). Legend: These plots are comparable with the results from the mixed ANOVA in Tables 3 and 4

Pairwise comparisons for the static standing tests are shown in Table 4. All patients showed increased sway in the back- and forward directions compared to the control group (Table 4). The lateral movement was significantly increased only in CM patients in comparison to healthy participants, whereas CM and LST patients showed significantly increased overall sway, as described by the *passed COP distance* (Table 4, Fig. 5). All presented sway parameters showed significantly increased motion with the eyes closed compared to the eyes opened (Table 4, Fig. 4).

**Table 4:** Pairwise comparison for static standing tests

		**Force distribution (AP)**	AP movement*	lateral movement*	Passed COP distance*
**NPH vs Control**	p	1.000	**.044**	1.000	.307
	Mean Difference [CI]	-3% [-11;5]	**2 mm [1;2.7]**	1.5 mm [0.4;2.7]	1.8 mm [1;2.7]
**CM vs Control**	p	1.000	**.001**	**.050**	**.008**
	Mean Difference [CI]	2% [-5;9]	**2.5 mm [1;2.7]**	**2.2 mm [1;7.4]**	**2.2 mm [1;7.4]**
**LST vs Control**	p	1.000	**.006**	.158	**.023**
	Mean Difference [CI]	-4% [-13;6]	**2.7 mm [1;7.4]**	2.5 mm [1;7.4]	**2.7 mm [1;7.4]**
**open vs closed eyes**	p	**.004**	**.001**	**.045**	**.001**
	Mean Difference [CI]	**-3% [-5;-1]**	**0.7 mm [0.4;1]**	**0.8 mm [0.4;1]**	**0.7 mm [0.4;1]**

^*^For ease of interpretation, the results of calculations and tests are back-transformed to their original scale. Significant values are given in bold

While CM patients showed the least balance parameter deviations with the eyes opened (Fig. 5), they showed stronger changes in balance parameters compared to the other groups with the eyes closed.

Overall, different patient groups showed distinct patterns of movement and balance parameters alterations compared to healthy controls, both for mean walking as well as balance parameters across trials (Fig. 1).

## Discussion

The presented results confirm the hypothesis that inertial measurement systems provide objective and quantifiable measures of different gait deviations in neurological patients. We established a setup of inertial sensors and pedobarographic measurements to collect the objective balance and kinematic motion data in an outpatient setting without the need for a clinical gait analysis laboratory. A laboratory-free motion analysis seems important, as the environment was previously found to significantly influence gait performance when comparing neurological patients under laboratory and real-world conditions [6]. Although the outpatient setting of the present study does not fully correspond to the real-world situation (which would require measurements to be obtained at home or outside in daily life), walking measurements obtained in a standardized in-door (large corridor) environment provided nearly unrestricted measurement conditions compared to those confined to a pure laboratory setting. Moreover, the wearable sensors used in our study proved to be easily applicable and easy to wear, not restricting the natural movement capacity of subjects in any way.

Comparison of different patient groups to a reference group of healthy controls in our study revealed different patterns of motion and balance parameter alterations across different gait disorders: Regarding motion parameters while walking, NPH patients showed significant differences in nearly all walking parameters compared to healthy controls, whereas fewer of the walking parameters differed in the lumbar stenosis group (affecting shoulder, sagittal hip, and transversal ankle ROM, as well as maximum ankle plantarflexion), and only some parameters (transverse hip, and transversal/ plantarflexion ankle ROM) being altered in the CM group. On the other hand, balance parameters showed the strongest deviations compared to healthy controls in the CM group (regarding AP movement, passed COP distance, and by trend lateral movement), followed by LST patients (regarding AP movement and passed COP distance), and least in the NPH group (affecting only AP movement) (please see Table 1). Pedobarographic measurements were furthermore found to be sensitive for different conditions (eyes opened or closed), showing significant effects on all balance parameters analysed especially in CM patients. Our findings of differently altered balance metrics across patient groups comply well with the clinical observation of sensory ataxia to be mainly expected in CM patients (due to involvement of the dorsal columns of the spinal cord), but not typically in NPH patients.

Overall, NPH, CM and LST patients showed distinct patterns of movement and balance parameter alterations compared to controls. As there are only few studies which actually used inertial measurement systems to compare different pathologies, our study extents current knowledge, in that it showed distinguishable patterns of movement and balance parameter alterations across different gait disorders, providing a trajectory for using inertial measurement systems and pedobarography for the objective and quantifiable discrimination of different pathologies. Advancing the application of such technologies may aid to increase diagnostic accuracy and to economize diagnostic procedures.

### NPH

The finding of a reduced ankle ROM in the transversal plane in our NPH patients may appear somewhat counterintuitive considering the clinically frequently observed outward rotation of the feet in NPH patients [34–36], but complies well with previous studies, in which restricted ankle movement was also reported in NPH patients [34]. By showing the overall rotation of the feet, we are aware that the outward placement of the feet is with respect to the line of progression in the transversal plane, and may arise from different levels (e.g. pelvis, hips, and ankles).

Arm swing has been described to be only mildly impaired in NPH patients, but has not been further analyzed in detail previously [34]. Our results show however a significantly decreased shoulder ROM in NPH patients compared to healthy controls (during one gait cycle by approximately 13°, Table 3), which may indicate a stronger hypokinetic impairment of the upper extremities in NPH patients than commonly noticed. The reduced hip ROM in the sagittal plane (during one gait cycle by approximately 10°) in our NPH patient group complies well with previous findings reported in the literature. Likewise, the reduced knee ROM or maximum knee flexion we found in NPH patients has been observed as well by other groups [34–36]. Both parameters were significantly decreased in NPH patients compared to CM patients, but not compared to LST patients. While it cannot be excluded, that the observed decreases in the ROM of different joints might be related to age effects rather than to specific gait disorders in our study, Stolz et al., previously described a decrease in ROM of the hip and knee in NPH patients, even compared to an age-matched reference group [34], which supports the results of the present study.

### LST

Kinematic movement parameter analyses in LST patients have repeatedly shown alterations particularly of lumbar and pelvic movement, with measurable impact of lower limb [26] and lower back pain [37] on movement patterns, and with objective and quantifiable measures of improvement after surgical intervention [28, 29]. One recent study [38] using optoelectronic techniques in a specialized laboratory setting described limited internal/external pelvic rotation and craniocaudal movement, limited hip extension and abduction/adduction, as well as limited ankle plantar flexion in this patient group. Our study confirms these previous findings in that the ROM of the hip and ankle were also restricted in our LST patients, while adding the observation of an additionally impaired ROM of the shoulders, which has not been reported before. In addition, we found balance metrics to be partially impaired in this patient group.

### CM

Concerning CM, kinematic movement data is also limited. In the literature, previous studies found significant differences in the knee ROM of CM patients compared to age-matched controls [21, 39–41], while another study found no significant impairment in the knee ROM during the stance phase [22], with the latter observation complying with our findings. Likewise, differing results have also been reported about the ROM of the ankle, which is increased in the sagittal plane in some studies [22, 39], while found to be decreased in another study [21], with the latter being as well supported by findings of our study. Such differences may in part relate to differences in the severity of clinical impairment among CM patients, as subclinical alterations of knee and plantarflexion have been reported in subclinical CM patients, as well as altered knee joint movement in severely affected CM patients (18), with an imbalance between agonist and antagonist muscles having been suggested as causative. CM patients previously showed aberrant sagittal alignment (with a larger anterior pelvic tilt and lumbar lordosis, but a lesser cervical lordosis and head flexion), impacting as well on biomechanics of the lower extremities (39). A previous study showed postoperative improvement in CM patients regarding balance, while movement parameters were not completely normalized (20). In another study, CM patients showed preoperatively greater range of motion of the ankle, the pelvis and the lumbar spine, but less ROM of the hip when compared to controls. Postoperatively, these patients showed increased knee and hip ROM, but lesser of the pelvis, the lumbar and cervical spine ROM (23), which may reflect regained postural stability after therapeutic intervention.

### Task conditions

Different study results might relate to differences in the measurement setup and data processing or might be grounded in the study design. Accordingly, Kuruvithadam et al. [6] showed, that environmental factors and measurement conditions may significantly alter motion patterns. Walking speed for example affects walking patterns [42], which is also visible in our results, as e.g. hip ROM significantly increased during fast walking compared to normal walking. On the other hand, the knee ROM is not significantly affected by the walking speed, supporting the hypothesis of a higher influence of walking speed on the hip than on knee motion. Malone et al. analysed CM patients in comparison to age-matched healthy participants walking at comfortable and matched walking speeds [21]. They found significantly reduced hip ROM in the sagittal plane only while walking at a comfortable speed, whereas a significantly reduced knee ROM in the sagittal plane was found for both walking speed conditions [21]. Therefore, specific gait disturbances may only manifest depending on gait velocity. While we did not measure gait velocity directly, we were able to identify the indirect effects of gait velocity on different metrics by comparing movement parameters obtained during normal and fast walking. Analysing different walking conditions seems essential given such findings, as it may increase the sensitivity for detecting specific gait deviations. Nevertheless, significant interaction effects concerning diagnosis groups and different walking tasks were only for the parameter *force distribution (AP)* during static standing across different conditions, which might relate to the lack of statistical power of the present study.

We expected the dual-task condition during walking to be indicative of cognitive impairment accompanying gait disorders. When comparing NPH to CM and LSK patients, we thus expected gait performance in NPH patients to be significantly more impaired during the dual task condition. As opposed to our expectations, however, current results showed no interaction effect in this regard, which might relate to the small sample size, but which we believe should be readdressed in future studies with larger samples.

### Future perspectives

While many instrumented gait analysis studies in patients with neurological gait disorders focuse on spatiotemporal metrics as such as walking speed, stride length, stride width, or cycle variables, extending movement analyses by kinematic movement parameters with the inclusion of the upper extremities may add to a better understanding of the complex pathophysiology of human locomotion. Integrating these findings may aid in more precisely phenotyping different movement disorders and might help to identify additional treatment targets for physical rehabilitation. Future studies should investigate larger patient samples not only cross-sectionally but also longitudinally, and instrumented motion and balance parameters may well be increasingly subjected to data-driven analyses, potentially enabling automated classification algorithms soon.

### Limitations

Although a multitude of different movement and balance parameters were analysed in the present study, the small and heterogenous sample size has to be regarded as a major limitation. Based on the small number of participants, we are well aware of the exploratory character of this study, whose findings need to be validated in further investigations. Furthermore, age differences across groups may be regarded as a further shortcoming, as we cannot exclude that some of the measured differences could be confounded by age effects, although normal gait characteristics of young and healthy subjects are commonly referred to in the literature. However, becauseof disease-immanent age differences of various gait disorders (per se limiting direct comparability of different patient groups with each other), we rather chose a common normative control group of young healthy subjects as “a common reference frame”, rather than choosing different control groups for each gait disorder. Moreover, the recruitment of “healthy elderly subjects” may not necessarily exclude the existence of some underlying, yet unrecognized pathology, which would rather distort the “healthy” reference values. Potentially age-related effects in our sample may even be smaller than suspected, as a study by Renggli et al. [14] showed differences between groups with a mean age difference of 50 years to be larger in a real-world environment, while age groups differed in a non-real-world environment only in stride and gait velocity. Moreover, age differences were larger than in our study so age effects might be further limited here.

As inertial sensors might be prone to various errors (signal drift, magnetic smog, movement of the sensors after calibration on the body segments), repeatability of measurements might be limited and will require further technical refinement and validation, although validity and reliability of inertial measurement unit-derived kinematics have been described as excellent for mean spatiotemporal parameters during walking [43] and with good to excellent agreement for all sagittal kinematic parameters when compared to optical motion capture systems [44]

Although the presented measurement setup is easily applicable, it may not be widely accessible to clinicians yet. Considering the complexity of human locomotion and the high dimensionality of parameters obtainable by instrumented gait and balance parameter measurements, it is necessary to further define those metrics most sensitive to neurological gait deviations, to economize data acquisition and minimize post-processing time. Furthermore, aspects of test–retest reliability, as well as measurement accuracy of the applied techniques, have to be addressed first, before they might be established in clinical routine.

## Conclusion

Our study showed that the application of inertial measurement systems and pedobarography is feasible in an outpatient setting in patients with different neurological gait disorders, showing advantages as a non-invasive, portable, fast, and easy-to-use examination device. This technique provided objective and quantifiable measures of differently altered motion and balance parameters across different patient groups. Further studies should however validate, whether inertial measurement systems and pedobarography allow to define specific profiles of movement and balance parameter patterns in the sense of specific ´gait signatures´, which could aid in increasing diagnostic accuracy in the discrimination of different pathologies as well as in disease monitoring, and in objectifying the clinical outcome after therapeutic intervention.

## Data Availability

The datasets used and/or analysed during the current study are available from the corresponding author on reasonable request.

## Abbreviations

NPH: Normal pressure hydrocephalus
CM: Cervicale myelopathy
LST: Lumbal stenosis
IMU: Inertial measurement unit
AP: Anterior-posterior
COP: Center of pressure
